# Process Monitoring for Vacuum-Assisted Resin Infusion by Using Carbon Nanotube-Based Sensors

**DOI:** 10.3390/polym17040459

**Published:** 2025-02-09

**Authors:** Yi Shi, Beibei Wang, Kui Du, Yanan Liu, Ruiqi Kang, Shaokai Wang, Jiayu Zhang, Yizhuo Gu, Min Li

**Affiliations:** 1 Key Laboratory of Aerospace Advanced Materials and Performance (Ministry of Education), School of Materials Science and Engineering, Beihang University, No. 37 Xueyuan Road, Haidian District, Beijing 100191, China; shiyi_1998@163.com (Y.S.); wangbeibei1001@126.com (B.W.); 15504151855@163.com (J.Z.); benniegu@buaa.edu.cn (Y.G.); 2 Advanced Material & Structure Laboratory, COMAC Beijing Aircraft Technology Research Institute, Future Science and Technology Park, Xiaotangshan, Changping District, Beijing 102211, China; dukui@comac.cc (K.D.); liuyanan@comac.cc (Y.L.); kangruiqi@comac.cc (R.K.)

**Keywords:** polymer composites, vacuum-assisted resin infusion, carbon nanotube, process monitoring, electrical resistance

## Abstract

This paper developed a carbon nanotube (CNT)-coated aramid fiber sensor, which was successfully used to monitor the resin flow front and sense the fluid pressure difference during the (VARI) process. The electrical resistance change of the CNT-coated fiber sensor was compared with that of buckypaper materials. The results show that the electrical resistances of CNT sensors show rapid growth successively along the infusion direction once the flow front reaches the sensor position during resin infusion in the VARI process. The electrical resistance of CNT-coated fiber sensors may increase by as much as 12 times after full impregnation. For the thicker preform, the resistance change Δ*R*/*R*_0_ of sensors on the top surface is closely related to fluid pressure, and bigger fluid pressure close to the inlet may result in a larger Δ*R*/*R*_0_. Two competitive factors affecting the electrical resistance of a CNT-coated sensor are revealed: aramid fiber tow swelling due to resin impregnation, and the compaction effect arising from resin pressure on the CNT network. In addition, the sensors on the top surface show a bigger Δ*R*/*R*_0_ than the bottom ones, and as the preform thickness decreases, these sensors tend to show smaller Δ*R*/*R*_0_.

## 1. Introduction

Vacuum-assisted resin infusion (VARI) is a popular liquid composite molding process. The reinforcement material is sealed and compacted using a flexible bag, and resin is driven to penetrate the porous preform under atmospheric pressure [1]. The external atmospheric pressure to compact the assembly is shared by the preform and resin system, and as the resin infusion proceeds, the preform may spring back, and its thickness changes [2]. The varied pressure distribution affects the preform compaction, permeability, and resin infusion. The monitoring of the resin flow front and fluid pressure has attracted a great deal of attention.

Many approaches have been introduced into the liquid composite molding process to monitor resin flow. Traditionally, the flow front movement may be captured via a flow visualization camera along with the time [3]. K. Spiridon developed an ultrasonic system to detect a transverse flow front and achieved the calculation of permeability [4]. C. Tuloup applied an in situ piezoelectric disk to monitor the whole manufacturing process of a glass fiber/polyester composite [5]. Various methods have been attempted to reduce the cost and the difficulty in operation.

Carbon nanomaterials, such as carbon nanotubes and graphene, possess excellent mechanical properties and piezoresistivity, which have been used for process monitoring [6], structural health monitoring [7,8,9], and so on. L. Zhang et al. prepared a Mxene/CNT buckypaper sensor to monitor the resin flow through a sandwich composite structure and calculated the permeability according to the monitored results [10]. S. Lu et al. prepared multi-walled CNT buckypaper by a spray vacuum method and successfully monitored the resin infusion into a glass fiber fabric with different permeability [11]. J. S. del Río et al. fabricated multi-walled carbon nanotube yarn which was integrated into different fabrics and successfully monitored the fluid front during VARI experiments [12]. J. R. N. Gnidakouong et al. coated multi-walled carbon nanotubes (CNTs) on a fiber textile to achieve in situ monitoring of infusion, crosslinking, and gel point during a liquid composite molding process [13]. G. Wang et al. prepared three types of carbon nanomaterial-based fiber sensors and also successfully monitored resin infiltration, gelation, cross-linking, and post-curing [14]. This research proved the feasibility of these conductive nanomaterials for flow front monitoring during liquid infiltration. However, the effect of complicated pressure distribution on the resistance response of these sensors is still unclear. W. N. Jeong monitored the thickness of carbon fiber composite during the VARI process by monitoring the variation in electrical resistance of a carbon fiber preform [15]. The CNT-based sensor has numerous tube-to-tube intersection points, which may better respond to the pressure difference for process monitoring.

This paper utilizes the swelling effect of a CNT-coated fiber sensor after resin impregnation to monitor the flow front and pressure difference. A CNT buckypaper sensor and a CNT-coated aramid fiber sensor were compared. The resistance response at different positions along the infusing direction and the through-thickness direction was investigated, and the sensitivity of the CNT sensor in the preform with different thicknesses was compared.

## 2. Materials and Methods

### 2.1. Materials

Two kinds of CNT-based sensors were compared in this study. CNT buckypaper was synthesized via a floating catalytic chemical vapor deposition (FCCVD) method, which was purchased from Suzhou Jiedi Nanotechnology Co., Ltd., Suzhou, Jiangsu Province, China. This buckypaper had a thickness of approximately 15 μm. Multi-walled CNT dispersion was purchased from Chengdu Organic Chemicals Co. Ltd., Chinese Academy of Sciences, Chengdu, China, and used to prepare a CNT-coated aramid fiber sensor. P-aramid fiber with a linear density of 444 dtex was supplied by China National Bluestar (Group) Co., Chengdu, China. EW100 plain-weave glass fabric was used to prepare the preform, which was supplied by Changzhou Bolong 3D Composite Materials Co., Ltd., Changzhou, China. The resin system of a HJ-966 epoxy and GC-966 curing agent, with a viscosity of approximately 0.4 Pa·s at ambient temperature, was chosen to infiltrate the fabric preform, which was supplied by Tianjin Dasen Material Science & Technology Co., Ltd., Tianjin, China. The auxiliary materials used in the VARI process were purchased from Airtech Advanced Materials Group, including bagging film, release film, peel ply, high-permeability medium, and sealant tape. Silver glue, used to connect wire and CNT-based sensors, was purchased from Shenzhen Luxianzi Technology Co., Ltd., Shenzhen, China.

### 2.2. Preparation of CNT-Based Sensors and the Sensor Arrangement in VARI Process

Two kinds of CNT-based sensors were prepared to monitor the VARI process, including CNT buckypaper and a CNT-coated aramid fiber sensor. The CNT buckypaper sensor was directly cut from a large sheet into 80 mm × 2 mm strips using a laser marking machine. The CNT-coated aramid fiber sensor was prepared by dripping a multi-walled CNT dispersion evenly along aramid fiber tow. The CNT dispersion penetrated into the fiber tow and the dripping process was stopped until the required mass of CNTs were coated on the aramid fiber. The mass ratio of CNT-to-aramid fiber was controlled at 10 wt%. The control of the CNT-to-fiber mass ratio was beneficial to the stable change of electrical resistance. The CNT-coated fiber sensor was further heated to 120 °C in a vacuum oven for 30 min to remove the residual solvent.

**Figure 1:**
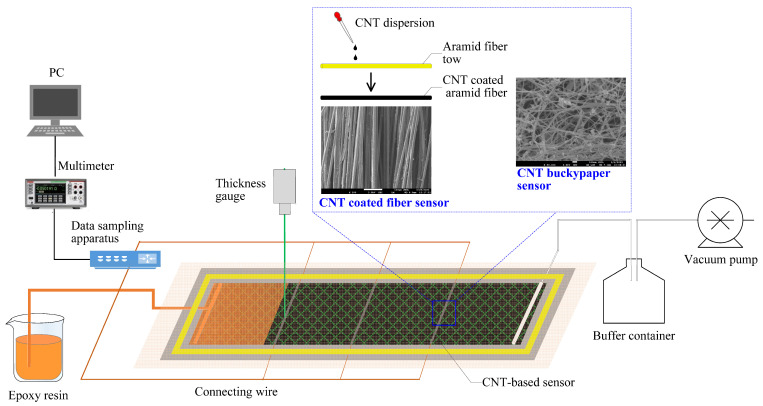
Schematic image of CNT-based sensor preparation and the monitoring application for VARI process.

In order to investigate the monitoring capability of these sensors for the VARI process, composite samples with the dimension of 40 cm × 8 cm were prepared with embedded CNT-based sensors, as shown in Figure 1. The sensor length was controlled at 8 cm, which was equal to the width of the fabric reinforcement. The enameled copper wire was used to connect these sensors with a Keithley DAQ6510 multimeter using conductive silver glue. Three pairs of sensors were placed perpendicular to the infusion direction, at 10, 20, and 30 cm away from the inlet, respectively. For each pair of sensors, they were separately placed on the top and bottom surfaces of the fabric reinforcement. After laminating peel ply and a high-permeability medium layer, the whole assembly was enclosed in a vacuum bag sealed on a mold plate. After the removal of trapped air inside the fiber was performed, an epoxy resin was penetrated into the preform under vacuum pressure. The electrical resistances of the CNT-based sensors were collected every second during the whole infusion process.

## 3. Results and Discussion

### 3.1. Electrical Resistance Variation of CNT-Based Materials After Resin Imbibition Freestandingly and in Closed Mold

CNT content plays a key role in the electrical resistance and resistance change [16,17]. In order to optimize the mass ratio of CNT-to-aramid fiber, CNT-coated fiber sensors with the mass ratios of 5 wt%, 10 wt%, 15 wt%, 25 wt%, 30 wt%, and 35 wt% were compared. Their electrical resistances were measured to be 9.23, 4.88, 4.29, 0.98, 0.94, and 0.83 kΩ, respectively. It can be seen that the electrical resistance decreased obviously in the mass ratio range of 5–10 wt%. This indicated that the variation of the CNT network in this mass ratio range may cause obvious resistance changes. This was also evidenced by the biggest temperature coefficient of resistivity for the CNT-coated fiber sensor with a mass ratio of 10 wt% during the heating process. As a result, a CNT content of 10% was determined to prepare the fiber sensor in the following study.

**Figure 2:**
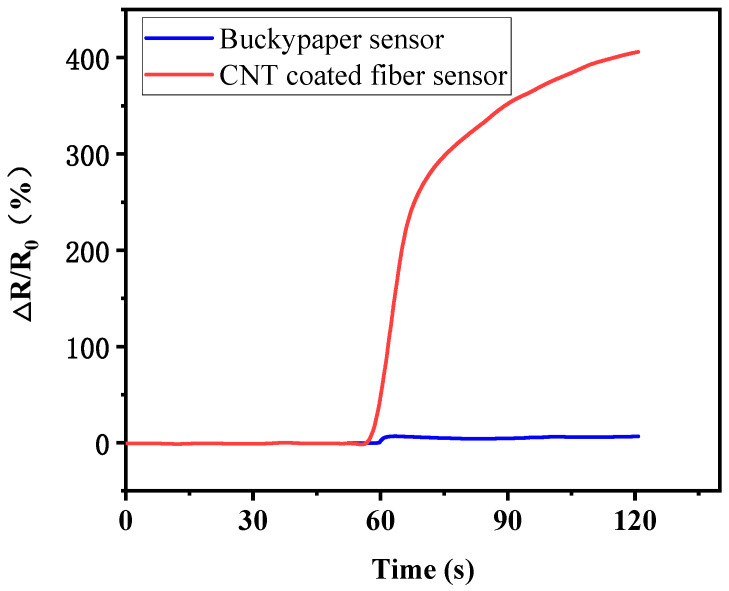
The electrical resistance variation of freestanding CNT-coated fiber and buckypaper material during resin imbibition.

Figure 2 shows the electrical resistance change (Δ*R*/*R*_0_) of CNT buckypaper strip and CNT-coated fiber during resin imbibition. Δ*R* is the difference between the electrical resistance before and after resin imbibition, and *R*_0_ is initial electrical resistance. Both the CNT-coated fiber and film material were freestandingly placed on an electric insulating mold, and epoxy resin was poured inside the mold along the side wall. The length between two measuring electrodes for both materials was approximately 80 mm, and the buckypaper material was cut into 2 mm strips. Once epoxy resin approached these CNT materials, the electrical resistance began to increase immediately. The CNT-coated fiber showed a much bigger electrical resistance change than the buckypaper materials. After these CNT materials were soaked in resin for 60 s, the Δ*R*/*R*_0_ value of the CNT-coated fiber reached as high as 400%, which was very sensitive to resin imbibition and showed great potential as sensors to detect resin flow. The Δ*R*/*R*_0_ value of the buckypaper material was 9%. The buckypaper material has a higher CNT content with a direct connection and overlapping of CNTs, which is unfavorable to a high Δ*R*/*R*_0_.

**Figure 3:**
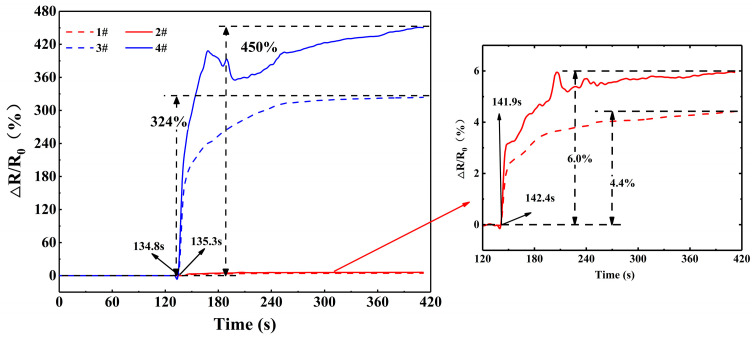
The electrical resistance variation of CNT-based sensors during resin infusion in resin transfer molding process.

In order to investigate the monitoring capacity of these CNT materials to resin infiltration, two CNT-coated fiber sensors were placed on the top and bottom surfaces of a glass fabric preform, perpendicular to the resin flow direction. The other two buckypaper sensors were also placed on the surfaces, which were parallel to the CNT-coated fiber sensors and farther from the inlet than the CNT-coated fiber sensors. The gauge length of these sensors was 80 mm, and the film sensor was also cut into 2 mm strips. The preform was compressed in a closed mold for the resin transfer molding process. Figure 3 shows the resultant electrical resistance variation of CNT-based sensors during liquid infusion. The inlet was opened at 130 s, and the resin system was used to penetrate the preform. It can be seen that both the CNT-coated fiber and buckypaper sensors were sensitive to resin flow, which showed increased electrical resistance sequentially during liquid penetration due to the smaller distance between the CNT-coated fiber sensors and the inlet. The sudden electrical resistance increase occurred at 134.8 and 135.3 s for the two CNT-coated fiber sensors, and the electrical resistance began to increase at 141.9 and 142.4 s for the two buckypaper sensors. Since liquid was infused from the preform edge, the electrical resistance change of these top and bottom sensors at the same distance from the inlet occurred almost at the same time. However, the top sensors showed a bigger Δ*R*/*R*_0_ value than the bottom ones for the same sensors. At 410 s, the Δ*R*/*R*_0_ values of the top and bottom CNT-coated fiber sensors reached 450% and 324%, respectively. The top and bottom buckypaper sensors showed Δ*R*/*R*_0_ values of 6.0% and 4.4%, respectively. Most of these sensors showed smaller Δ*R*/*R*_0_ values in the closed mold than that during the freestanding resin imbibition process. In addition, the buckypaper sensor with a larger aspect ratio showed a smaller Δ*R*/*R*_0_ value compared with the previous results [18].

### 3.2. Electrical Resistance Variation of CNT-Based Sensors During Resin Infusion in VARI Process

**Figure 4:**
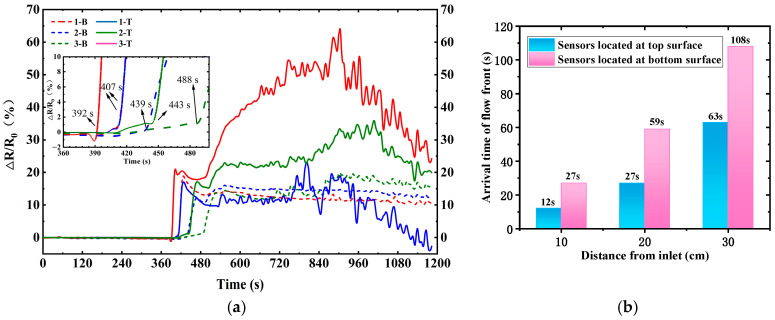
(**a**) The electrical resistance variation of CNT buckypaper sensor with the inlet opening at 380 s, and (**b**) the arrival time of flow front at different sensor positions during resin infusion into fabric reinforcement with 12 plies of EW100 glass fabric.

Figure 4 shows the electrical resistance variation (Δ*R*/*R*_0_) of the CNT buckypaper sensor during resin infusion into the preform consisting of 12 plies of glass fabric in the VARI process. These sensors on the top surface of the preform are denoted as 1-T, 2-T, and 3-T from inlet to outlet, and those at the bottom surface are denoted as 1-B, 2-B, and 3-B. To ensure the stability of the whole monitoring system, the preform with embedded sensors was compacted under vacuum for at least 5 min after the beginning of the electrical resistance data collection.

CNT buckypaper has demonstrated its great piezoresistivity [18], and the long nanotubes and dense network of FCCVD film result in its superior electrical conductivity. The electrical resistance of the 80 mm × 2 mm pristine CNT buckypaper sensor was measured to be approximately 90 Ω. As seen in Figure 4, after the inlet is opened at 380 s, the electrical resistances of these sensors show rapid growth successively along the infusion direction. The nanoscale pores and big specific surface area of the CNT buckypaper result in high capillary pressure and good adsorption capacity, and thus the quick absorption of epoxy resin causes a rapid increase in intertube resistance. The maximum electrical resistance variation (Δ*R*/*R*_0_) values reach 64%, 23%, and 35% for the 1-T, 2-T, and 3-T sensors, respectively. The maximum Δ*R*/*R*_0_ values of 1-B, 2-B, and 3-B at the bottom surface are also bigger than 10% after resin impregnation. Figure 4b further shows the arrival time of the flow front. The turning point of the Δ*R*/*R*_0_ curve from the stabilized electrical resistance at the beginning to the rapid increase is defined to be the arrival time of the flow front. The monitored arrival times for 1-T, 2-T, and 3-T are 12, 27, and 63 s, respectively, which are consistent with the observation of the infusion process. During the VARI process, epoxy resin infuses into the high-permeability medium preferentially and then penetrates the fiber preform through the thickness direction. The monitored arrival times for 1-B, 2-B, and 3-B are 27, 59, and 108 s, respectively, which are behind the arrival times of the corresponding sensors on the top surface, as expected. This indicates the capability of the CNT buckypaper sensor for the flow front monitoring of the VARI process. Although this kind of CNT buckypaper sensor showed a smooth Δ*R*/*R*_0_ in the resin transfer molding process in previous research [19], the Δ*R*/*R*_0_ curve in the current study showed large fluctuations during the VARI process, which should be related to the varied resin pressure.

**Figure 5:**
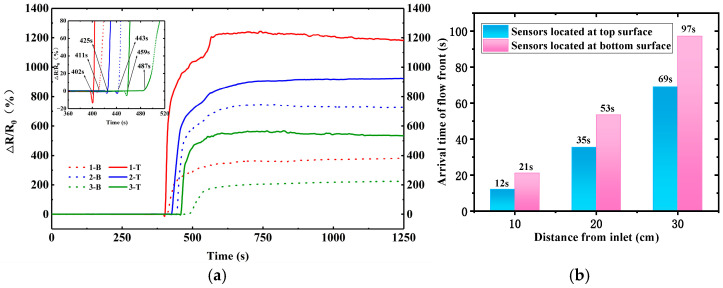
(**a**) The electrical resistance variation of CNT-coated fiber sensor with the inlet opening at 390 s, and (**b**) the arrival time of flow front at different sensor positions during resin infusion into reinforcement with 12 plies of EW100 glass fabric.

Figure 5 shows Δ*R*/*R*_0_ and time curves of CNT-coated fiber sensors during the VARI process. Twelve plies of glass fabric were laminated to prepare the preform. The initial electrical resistance of the CNT-coated fiber sensor is approximately 5 kΩ, which is much bigger than that of the buckypaper sensor. Accompanied by the arrival of the flow front, the electrical resistances of these sensors increase dramatically. The inlet is opened at 390 s, and the monitored arrival time intervals from the inlet opening are 12, 35, 69, 21, 53, and 97 for the 1-T, 2-T, 3-T, 1-B, 2-B, and 3-B sensors. It can be seen that the electrical resistance responds successively for these sensors from the inlet to the outlet direction following the infusion process, and the response of the bottom sensor is behind the corresponding top sensor, as expected. The arrival times of the flow front for the CNT-coated fiber sensor (Figure 5b) and the film sensor (Figure 4b) are close. In order to ensure the reliability of the CNT-coated fiber sensor, the infusion experiment was repeated three times. The coefficient of variation for the arrival time was no more than 8%. Since the deviation may be caused by the sensor and fabric preform, the arrival time results in one typical experiment were presented.

In addition to the sensitivity to the flow front, CNT-coated fiber sensors yield smooth Δ*R*/*R*_0_ curves. Especially, the maximum electrical resistance variation (Δ*R*/*R*_0_) values reach as high as 1240%, 920%, and 566% for the 1-T, 2-T, and 3-T sensors, respectively, which are much bigger than those of buckypaper sensors. These results show the stronger sensitivity and better stability of the CNT-coated fiber sensor to and under resin impregnation. Table 1 lists the Δ*R*/*R*_0_ of different sensors for the monitoring of resin infusion. It can be seen that the CNT-coated fiber sensor shows great sensitivity compared with other sensors.

The electrical resistance of the CNT-coated sensor is influenced by two competitive factors: aramid fiber tow swelling due to resin impregnation, and a compaction effect arising from resin pressure. For the CNT-coated sensor, the aramid fiber bundle is coated by entangled CNTs. During the VARI process, the applied air pressure on the whole system is undertaken by the preform and resin together. As the resin system penetrates into the fiber bundle, the compaction load applied on the fiber bundles become smaller, and the fiber bundles may swell because the resin partially absorbs the external pressure. The swelling of the fiber bundles may easily break the interaction between these entangled CNTs, which may increase the electrical resistance due to the change of contact resistance. On the other hand, before resin infiltration, the CNT network is locally compacted due to the limited contact with porous fiber reinforcement, especially for these top sensors. After resin impregnation, the external pressure is thoroughly applied on the CNT network through resin and the contact preform, which may cause the decrease in the CNT’s intrinsic electrical resistance and tube-to-tube contact resistance. For these top sensors on 12 plies of glass fabric, the swelling effect plays a key role in resistance change. The more obvious swelling at the high-resin-pressure zone near the inlet results in a bigger resistance increase. This indicates that the CNT-coated fiber sensor may detect the pressure difference for the VARI process. Also, the bottom sensors show smaller Δ*R*/*R*_0_ values than the top sensors. This is because these bottom sensors are fully in contact with the solid mold during the whole process, and the microstructure evolution of the bottom sensors is inhibited.

Borrowing ideas from the swelling and shrinking of gels, the swelling of the CNT-based sensor during resin infusion may be considered to consist of two consecutive processes: pure diffusion and a shear relaxation process [20,21]. For the CNT-coated fiber sensor, the CNT length is smaller than 10 μm, while the buckypaper material synthesized via the FCCVD method consists of entangled millimeter-long CNTs. The smaller aspect ratio of CNTs in the CNT-coated fiber sensor resulted in a smaller relaxation time than the buckypaper sensor. Thus, the CNT-coated fiber sensor reached a stable electrical resistance more quickly.

### 3.3. Effect of Preform Thickness on the Sensitivity of CNT-Coated Fiber Sensor

**Figure 6:**
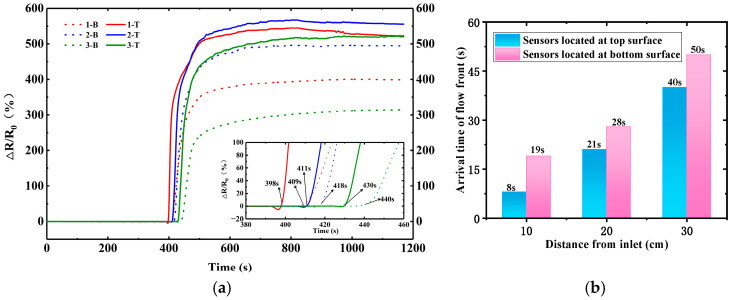
(**a**) The electrical resistance variation of CNT-coated fiber sensor with the inlet opening at 390 s, and (**b**) the arrival time of flow front at different sensor positions during resin infusion into reinforcement with 6 plies of EW100 glass fabric.

Figure 6 shows the resistance change of CNT-coated fiber sensors during resin infusion into the preform consisting of six plies of glass fabric. These sensors show the same response sequence to the flow front as in Figure 5. The monitored arrival time intervals from the inlet opening are 8, 21, 40, 19, 28, and 50 for the 1-T, 2-T, 3-T, 1-B, 2-B, and 3-B sensors, which are smaller than those in 12 plies of glass fabric. The response time of these sensors is consistent with the visual observation of the flow front. Compared with the results in Figure 5, most of those sensors inside a thinner preform show smaller Δ*R*/*R*_0_ values. Meanwhile, the 2-T and 2-B sensors show the biggest Δ*R*/*R*_0_ values among the top and bottom sensors, respectively. A similar phenomenon is observed for these bottom sensors in Figure 5 with 12 plies of glass fabric, which should be the competition result of fiber tow swelling and CNT network compaction. It should be pointed out that Δ*R*/*R*_0_ does not show an obvious response after the close of the inlet.

**Figure 7:**
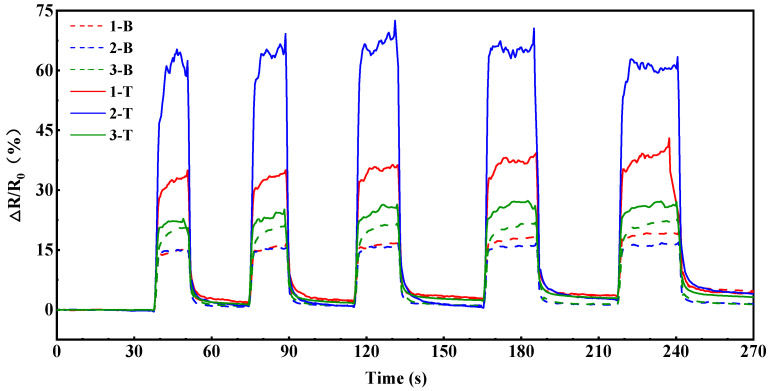
The electrical resistance variation of CNT-coated fiber sensor during loading and unloading vacuum.

In order to better understand the interior mechanism of bigger Δ*R*/*R*_0_ values during the resin infusion of the VARI process than during the resin transfer molding and resin wicking process for the same sensors, multiple cycles of loading and unloading vacuum pressure on a fiber preform were carried out before resin infusion. CNT-coated fiber sensors were placed on the surface of 6 plies of EW100 glass fabric, as in Figure 1, and a vacuum was maintained for 10 min before the measurement. The vacuum was applied five times repeatedly and maintained for 15–20 s. Figure 7 shows the resistance change of CNT-coated fiber sensors during preform compaction and the springback process. It can be observed that the electrical resistance change of the same sensor was close during the five cycles, indicating the stable response of these sensors. After the vacuum pressure was unloaded, the electrical resistance increased and was stabilized in a certain range rapidly for each time. When the vacuum was drawn again, the electrical resistance decreased rapidly, but it took a longer time to recover to the initial electrical resistance. This indicated that the fiber fabric was compacted gradually. The sensors on the top surface close to the vacuum bag also showed bigger Δ*R*/*R*_0_ values than those close to the mold after the springback of dry fabric. Among these sensors, the Δ*R*/*R*_0_ value may reach 60% after the springback of dry fabric.

**Figure 8:**
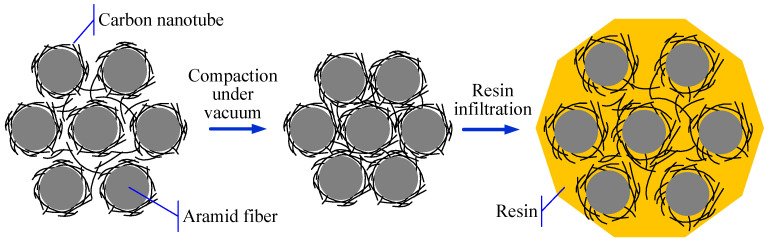
The mechanism of electrical resistance variation for CNT-coated fiber sensor during VARI process.

According to the analysis of electrical resistance during fabric springback and resin infusion, the variation mechanism was drawn in Figure 8. During the VARI process, both the fabric and sensor were compacted at the beginning, which increased the tube-to-tube contact, and the initial electrical resistance was maintained at a smaller value than for the pristine sensor. This was beneficial to the amplification of the electrical resistance response in the following resin infusion process. When resin impregnated the preform and the CNT sensor, the compacted sensor sprang back, which reduced the CNT volume fraction. The infused resin may also reduce the tube-to-tube contact resistance. As a result, the CNT-coated fiber sensor showed a significant electrical resistance change during the VARI process. The different degree of fabric springback at different positions provided the capability of the CNT-coated fiber sensor for pressure monitoring.

## 4. Conclusions

This paper developed a CNT-coated aramid fiber sensor and investigated its electrical resistance response to flow front and pressure during the VARI process by comparing it with a buckypaper sensor. During resin infusion in the VARI process, the electrical resistances of the CNT sensors show rapid growth successively along the infusion direction once the flow front reaches the sensor position. The electrical resistance of the CNT sensor may increase by as much as 12 times after full impregnation, which is much higher than for film material. For the thicker preform, the Δ*R*/*R*_0_ of the top sensors is closely related to fluid pressure, and the bigger fluid pressure close to the inlet may result in a larger Δ*R*/*R*_0_. Two competitive factors are revealed to affect the electrical resistance of the CNT-coated sensors: aramid fiber tow swelling due to resin impregnation, and the compaction effect on the CNT network arising from resin pressure. In addition, the top sensors show a bigger Δ*R*/*R*_0_ than the bottom ones. As the preform thickness decreases, most sensors show a smaller Δ*R*/*R*_0_.

## Figures and Tables

**Table 1:** Comparison of Δ*R*/*R*_0_ for different sensors during the monitoring of resin infusion.

Type of Sensor	Maximum Δ*R*/*R*_0_	References
MXene/CNT sensor	~11.5	[10]
Filtered buckypaper sensor	~2.5	[11]
MWCNT yarn	~0.08	[12]
MWCNT-spray-coated fiber textile	~1.8	[13]
Carbon fiber fabric	<0.6	[15]
CNT-coated aramid fiber sensor	12.4	This study

## Data Availability

The original contributions presented in this study are included in the article, further inquiries can be directed to the corresponding authors.
